# Day Ahead Optimal Dispatch Schedule in a Smart Grid Containing Distributed Energy Resources and Electric Vehicles

**DOI:** 10.3390/s21217295

**Published:** 2021-11-02

**Authors:** Maria Fotopoulou, Dimitrios Rakopoulos, Orestis Blanas

**Affiliations:** Chemical Process and Energy Resources Institute, Centre for Research and Technology Hellas, 52 Egialias Str., Maroussi, GR-15125 Athens, Greece; fotopoulou@certh.gr (M.F.); blanas@certh.gr (O.B.)

**Keywords:** smart grid, V2G, day ahead optimization, energy management, distributed energy resources

## Abstract

This paper presents a day ahead optimal dispatch method for smart grids including two-axis tracking photovoltaic (PV) panels, wind turbines (WT), a battery energy storage system (BESS) and electric vehicles (EV), which serve as additional storage systems in vehicle to grid (V2G) mode. The aim of the day ahead schedule is the minimization of fuel-based energy, imported from the main grid. The feasibility of the proposed method lies on the extensive communication network of the smart grids, including sensors and metering devices, that provide valuable information regarding the production of the distributed energy resources (DER), the energy consumption and the behavior of EV users. The day ahead optimal dispatch method is applied on a smart grid in order to showcase its effectiveness in terms of sustainability, full exploitation of DER production and ability of EVs to act as prosumers.

## 1. Introduction

The reduction of greenhouse gas emissions is a primary goal of the European Union that has already produced encouraging results [1]. More specifically, as part of the European Green Deal, the European Commission has set the target of 55% reduction by 2030 in greenhouse gas emissions compared to 1990 [2]. Toward that end, the adoption of renewable energy sources (RES) such as photovoltaic panels (PV) and wind turbines (WT), as well as battery energy storage systems (BESS) and other energy storage systems (ESS), is ever increasing [3,4,5]. Another recent trend is the procurement of electric vehicles (EVs) by public and private entities alike [6,7,8]. The importance of the mass use of EVs is paramount as they reduce greenhouse gas emissions compared to conventional vehicles using fossil fuels [9]. Though electric vehicles are usually considered as loads to the grid since they are charged from it (G2V), they also have the capability of acting as power sources that can give back energy from the vehicle to the grid (V2G) [10,11]. Essentially, this allows electric vehicles to act as small BESSs for the duration of their stay at a charging station, which increases the flexibility of the grid and the capability for RES penetration [12,13,14].

The aforementioned technologies can be efficiently combined in smart grids [15,16]. A defining characteristic of smart grids aside from “green energy” presence is that they contain advanced metering infrastructure (AMI) comprising smart sensors and meters capable of both measurements and wireless communication with other devices to use all the integrated assets to their full potential [17,18]. For example, smart grids employ smart sensors and smart meters to determine when EVs arrive and depart, the state of charge of the EVs and grid BESSs, the angles of the PVs to implement tracking for optimal generation, etc. [19]. The integration of all these sensors makes smart grids exemplary cyber-physical systems that allow flow of information among their assets. The data from sensors is provided securely through communication protocols to the operator so that they can take the necessary actions and can then be stored, to potentially make predictions for the future based on them. Of note, this sort of environment also promotes the incorporation of more recent, advanced technologies in the overall monitoring and control system, such as the internet of things (IoT), home energy management, etc. [20,21,22,23].

All the available information by the smart sensors and smart meters to the operator make day-ahead optimal scheduling possible [24,25,26], especially in combination with forecasting techniques, EV charging patterns, solar and wind databases, etc. [27,28,29]. This scheduling is extremely useful for the grid operator that can estimate the dispatch required to satisfy consumers and the charge and discharge schedule of EVs and BESSs, doing so in a manner that maximizes a desired benefit. Thus, day-ahead optimization provides a benchmark regarding the schedule of distributed energy resources (DER) utilization, without which the energy dispatch decisions would have to be made on-the-spot (which would be quite insufficient, especially in the case of V2G, where the dispatch needs to be conducted according to the schedules of the EV owners) [30,31,32]. Of course, day-ahead scheduling is not without its flaws as there are errors in all its included predictions. So, it is imperative that the errors have a known bound and that their impact is taken into account [33].

Several approaches have been proposed in literature to address day-ahead optimization problems for smart grids that include EVs with V2G mode and RES production. They differ from each other in their goals and the assets they add to the installation under study. For example, in [34] an optimization model is introduced for a grid including both EVs that support V2G mode and PV panels. The purpose of the optimization is the maximization of self-consumption. Additionally, in [35] a similar installation is investigated, including both EVs with V2G capability and PV panels, but this time the objective is to minimize the cost for the grid operator while also minimizing the load variance throughout the day. The optimization is achieved through particle swarm optimization (PSO), which is a meta-heuristic method. However, there are cases where the integration of EVs (considering V2G mode) in a grid is studied along with wind farms (WFs), instead of PV panels, as presented the work of [36,37,38,39]. More specifically, in the case of [36] the objective is to simultaneously optimize the wind power curtailment, the generation cost, and the emissions from the daily operation of the grid. Additionally, in [37] the goal of the proposed methodology is to achieve high wind integration and low charging cost for the EV users. The purpose of [38,39] is to minimize the cost for the operator through the optimal charge/discharge schedule of the EVs in combination with the wind generation. Furthermore, there are studies where the EVs are combined with both PVs and wind generation. For example, in [40] an optimization model for microgrids (MG) including wind and solar generation as well as EVs is introduced. The purpose of the optimization is to provide the EV’s charging/discharging schedule that minimizes the variance of equivalent load, considering a rich environmental-friendly energy mix. Additionally, research has been conducted regarding the operation of EVs (including V2G mode) in grids that incorporate a variety of DER, as presented in [41,42]. More specifically, [41] proposes an optimization model for a grid that includes EVs, PV installations, wind generation, a diesel engine and a microturbine. The authors utilize two variations of PSO to generate an optimal day-ahead schedule in terms of cost minimization and maximization of environmental protection. Furthermore, the authors of [42] study a grid that consists of EVs, a PV installation, a WF and a gas turbine. The purpose of the study is to minimize the system compensation and abandoned energy costs while maximizing distributed generation income. There are also cases where the aspect studied is not the viewpoint of the smart grid operator but the one of the operator of the EV station, as presented in the work of [43,44]. In fact, the authors of [43] solve the dispatch problem from the side of the operator of an EV charging station, where the EVs support V2G mode. The station is equipped with a PV installation and a BESS. The purpose of the day-ahead optimization is to maximize the daily profit of the station and minimize the losses in the dedicated BESS lifetime. In a similar installation, in [44], the operator of an EV charging station equipped with PV panels and an ESS aims to minimize the total net cost of charging vehicles and maximize V2G penetration, PV power sale, and ESS usage.

**Figure 1:**
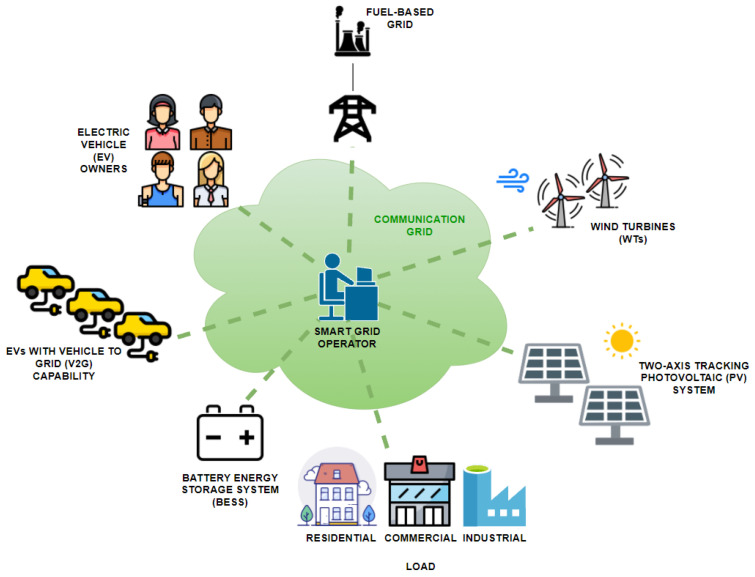
Smart grid including a variety of DER and a communication grid.

The present paper proposes a day-ahead scheduling method, taking advantage of the extensive communication network of the smart grids (sensors, metering devices, etc.) which promote the efficient integration of a variety of DER and EVs. The problem includes an extensive number of smart grid assets: a main (fuel-based) grid, a PV system with two-axis tracking, a WF, a BESS, industrial, commercial and residential loads and a large number (300) of EVs with V2G capability, as presented in Figure 1. Since the proposed methodology takes into consideration all of these environmental-friendly assets (instead of a subset of them), it expands the scope of the [34,35,36,37,38,39,40,41,42,43,44], thus contributing to the research field of environmental-friendly technologies and smart grid energy dispatch. Furthermore, due to environmental concerns (and the presence of RES and storage units), the objective is to minimize the environmental footprint of the smart grid, but attention is also given to cost factors where possible as the paper takes into account the reduced electricity price offered to consumers during certain hours of the day in Greece (reduced grid price schedule, [45]). According to the approach of the proposed methodology, the EVs and the BESS are charged with energy produced by the smart grid’s RES. In addition to the main contribution, described above, it is noted that the BESS never discharges completely to ensure that the grid always has a quantity of energy available to it (back-up) regardless of circumstances. Furthermore, the problem is solved so that parity is maintained between all controllable assets (main fuel-based grid, BESS and EVs). The results of the proposed methodology are compared with the respective results without V2G capability, but also with the results of an extensive sensitivity analysis. To conclude with, the main contribution of this paper is:

1. The optimization of the energy management in a smart grid that includes a PV system, a WF, a BESS and EVs with V2G capability.
2. The minimization of energy exchange between the fuel-based main grid and the smart grid and the maximization of the smart grid’s self-consumption. Thus, the EVs and the BESS are charged from RES.
3. The reduced electricity prices are taken into account.

The paper is organized as follows: Section 2 describes the methodology (formulation of the optimizer and uncertainties related to day-ahead optimization), Section 3 describes the case study, Section 4 presents the results of the case study, but also the results of the sensitivity analysis, while Section 5 presents the conclusions.

## 2. Methodology

### 2.1. Formulation of the Optimizer

The basis of the proposed day-ahead optimization method constitutes a mixed-integer linear programming (MILP) model, since it includes both continuous and binary variables. Its scope is to optimally utilize the RES of the smart grid (i.e., the two-axis tracking PV system and the WTs), the EVs (considering their potential to act as both programmable loads and storage units) and the BESS, in order to minimize the energy exchange between the fuel-based main grid and the smart grid and maximize the smart grid’s self-consumption. The day-ahead optimization requires forecasts of the PV and WF production as well as forecasts regarding the behavior of EV owners. It does also require information regarding the technical specifications of the smart grid’s assets (e.g., the efficiency of the EVs and the BESS). The outcome is a twenty-four-hour schedule composed of the optimal charge/discharge actions for each controllable asset (i.e., the EVs and the BESS).

The objective function (1) minimizes the environmental footprint of the smart grid by minimizing the fuel-based energy imported from the main grid and the energy exported to it. $I_{t}$ is the power imported from the main grid at time-step t. $E_{t}$ is the power exported to the main grid at time-step t. $\Delta_{t}$ is the duration of the time-step. It is noted that there are 24 time-steps, one for each hour of the day.

(1) $$\mathrm{minF}=\overset{24}{\underset{t=1}{\sum }}{(I}_{t}\Delta_{t}{+E}_{t}\Delta_{t})$$

The energy balance of the smart grid at time-step t is represented by (2). $P_{t}^{\mathrm{PV}}$ is the two-axis tracking PV production at time-step t. $P_{t}^{\mathrm{WF}}$ is the WF’s generation at time-step t. $D_{t}^{{\mathrm{EV}}_{i}}$ is the power discharged from the i-th EV at time-step t. $D_{t}^{\mathrm{BESS}}$ is the power discharged from the smart grid’s BESS at time-step t. $L_{t}$ is the load of the smart grid at time-step t. $C_{t}^{{\mathrm{EV}}_{i}}$ is the power charged to the i-th EV (or ${\mathrm{EV}}_{i}$) at time-step t. $C_{t}^{\mathrm{BESS}}$ is the power charged to the smart grid’s BESS at time-step t.

(2) $$P_{t}^{\mathrm{PV}}\Delta_{t}{+P}_{t}^{\mathrm{WF}}\Delta_{t}+\underset{i}{\sum }{(D}_{t}^{{\mathrm{EV}}_{i}}\Delta_{t}{)+D}_{t}^{\mathrm{BESS}}\Delta_{t}{+I}_{t}\Delta_{t}{\;-\;E}_{t}\Delta_{t}{=L}_{t}\Delta_{t}+\underset{i}{\sum }{(C}_{t}^{{\mathrm{EV}}_{i}}\Delta_{t}{)+C}_{t}^{\mathrm{BESS}}\Delta_{t},\;\forall t$$

The upper and lower limits of the state of charge of each EV, ${\mathrm{SOC}}_{t}^{{\mathrm{EV}}_{i}}$, are expressed by (3), where ${\mathrm{SOC}}_{\min}^{{\mathrm{EV}}_{i}}$ is the minimum and ${\mathrm{SOC}}_{\max}^{{\mathrm{EV}}_{i}}$ is the maximum state of charge respectively. As expressed by (4), at the time of departure of each EV, ${\mathrm{Tdep}}_{i}$, the state of charge needs to be equal to the user-defined one, ${\mathrm{USOC}}_{\;}^{{\mathrm{EV}}_{i}}$.

(3) $${\mathrm{SOC}}_{\min}^{{\mathrm{EV}}_{i}}\;\leq {\;\mathrm{SOC}}_{t}^{{\mathrm{EV}}_{i}}\;\leq {\;\mathrm{SOC}}_{\max}^{{\mathrm{EV}}_{i}},\;\forall t,i$$

(4) $${\mathrm{SOC}}_{{t=\mathrm{Tdep}}_{i}}^{{\mathrm{EV}}_{i}}{=\mathrm{USOC}}_{\;}^{{\mathrm{EV}}_{i}},\;\forall i$$

The energy balance of each EV is expressed by (5) where $\eta^{{\mathrm{EV}}_{i}}$ is the efficiency of each EV’s battery and $T_{t}^{{\mathrm{EV}}_{i}}$ is the consumption rate due to transportation. Of course, an EV can only be charged or discharged when it is parked, as expressed by (6) and (7) respectively.

(5) $${\mathrm{SOC}}_{t+1}^{{\mathrm{EV}}_{i}}{=\mathrm{SOC}}_{t}^{{\mathrm{EV}}_{i}}{+C}_{t}^{{\mathrm{EV}}_{i}}\eta^{{\mathrm{EV}}_{i}}\Delta_{t\;}-\;\frac{D_{t}^{{\mathrm{EV}}_{i}}\Delta_{t}}{\eta^{{\mathrm{EV}}_{i}}}\;{-\;T}_{t}^{{\mathrm{EV}}_{i}}\Delta_{t},\;\forall t,i$$

(6) $$C_{t}^{{\mathrm{EV}}_{i}}{=0\;\mathrm{if}\;T}_{t}^{{\mathrm{EV}}_{i}}\;>\;0,\;\forall t,i$$

(7) $$D_{t}^{{\mathrm{EV}}_{i}}{=0\;\mathrm{if}\;T}_{t}^{{\mathrm{EV}}_{i\;}}>\;0,\;\forall t,i$$

The maximum power that can be charged to an EV, $C_{\max}^{{\mathrm{EV}}_{i}}$, is taken into account in (8). It is noted that $u_{{\mathrm{ch}}_{t}}^{{\mathrm{EV}}_{i}}$ is the binary variable that indicates whether the i-th EV is being charged at time-step t or not. Similarly, the maximum power that can be discharged from an EV, $D_{\max}^{{\mathrm{EV}}_{i}}$, is taken into account in (9), where $u_{{\mathrm{dch}}_{t}}^{{\mathrm{EV}}_{i}}$ is the binary variable that indicates whether the i-th EV is being discharged at time-step t or not.

(8) $$C_{t}^{{\mathrm{EV}}_{i}}\;\leq {\;C}_{\max}^{{\mathrm{EV}}_{i}}u_{{\mathrm{ch}}_{t}}^{{\mathrm{EV}}_{i}},\;\forall t,i$$

(9) $$D_{t}^{{\mathrm{EV}}_{i}}\;\leq {\;D}_{\max}^{{\mathrm{EV}}_{i}}u_{{\mathrm{dch}}_{t}}^{{\mathrm{EV}}_{i}},\;\forall t,i$$

Additionally, as expressed by (10), an EV cannot be simultaneously charged and discharged.

(10) $$u_{{\mathrm{ch}}_{t}}^{{\mathrm{EV}}_{i}}{+u}_{{\mathrm{dch}}_{t}}^{{\mathrm{EV}}_{i}}=1,\;\forall t,i$$

Regarding the smart grid’s BESS, it is noted that in order to have back-up in case of an emergency a certain quantity of energy needs to always be stored in it [46]. Therefore, the minimum acceptable state of charge of the BESS is not the technical minimum but the one that ensures the grid’s seamless operation in case of an emergency. This value is expressed by the parameter ${\mathrm{SOC}}_{\min}^{\mathrm{BESS}}$ and along with the maximum state of charge of the smart grid’s BESS, ${\mathrm{SOC}}_{\max}^{\mathrm{BESS}},\;$limits the BESS’s state of charge, ${\mathrm{SOC}}_{t}^{\mathrm{BESS}}$, as presented in (11).

(11) $${\mathrm{SOC}}_{\min}^{\mathrm{BESS}}\;\leq {\;\mathrm{SOC}}_{t}^{\mathrm{BESS}}\;\leq {\;\mathrm{SOC}}_{\max}^{\mathrm{BESS}},\;\forall t$$

The energy balance of the BESS is expressed by (12) where $\eta^{\mathrm{BESS}}$ is the BESS’s efficiency.

(12) $${\mathrm{SOC}}_{t+1}^{\mathrm{BESS}}{=\mathrm{SOC}}_{t}^{\mathrm{BESS}}{+C}_{t}^{\mathrm{BESS}}\eta^{\mathrm{BESS}}\Delta_{t}\;-\;\frac{D_{t}^{\mathrm{BESS}}\Delta_{t}}{\eta^{\mathrm{BESS}}},\;\forall t$$

The maximum power that can be charged to the BESS, $C_{\max}^{\mathrm{BESS}}$, is taken into account in (13). It is noted that $u_{{\mathrm{ch}}_{t}}^{\mathrm{BESS}}$ is the binary variable that indicates whether the BESS is being charged at time-step t or not. Similarly, the maximum power that can be discharged from the BESS, $D_{\max}^{\mathrm{BESS}}$, is taken into account in (14), where $u_{{\mathrm{dch}}_{t}}^{\mathrm{BESS}}$ is the binary variable that indicates whether the BESS is being discharged at time-step t or not.

(13) $$C_{t}^{\mathrm{BESS}}\;\leq {\;C}_{\max}^{\mathrm{BESS}}u_{{\mathrm{ch}}_{t}}^{\mathrm{BESS}},\;\forall t$$

(14) $$D_{t}^{\mathrm{BESS}}\;\leq {\;D}_{\max}^{\mathrm{BESS}}u_{{\mathrm{dch}}_{t}}^{\mathrm{BESS}},\;\forall t$$

Finally, as expressed by (15), the BESS cannot be simultaneously charged and discharged.

(15) $$u_{{\mathrm{ch}}_{t}}^{\mathrm{BESS}}{+u}_{{\mathrm{dch}}_{t}}^{\mathrm{BESS}}=1,\;\forall t$$

Regarding the smart grid’s wind generation, it is noted that the total WF generation at time-step t, $P_{t}^{\mathrm{WF}}$, consists of the sum of each WT’s generation at time-step t, $P_{t}^{{\mathrm{WT}}_{w}}$, where w is the index of each WT, as presented in (16):

(16) $$P_{t}^{\mathrm{WF}}=\overset{\mathrm{WTs}}{\underset{\;}{\sum }}P_{t}^{{\mathrm{WT}}_{w}},\;\forall t$$

Regarding the smart grid’s load at time-step t, $L_{t}$, all three possible load types are considered, i.e., residential, $L_{t}^{R}$, commercial, $L_{t}^{C}$, and industrial, $L_{t}^{I}$, as presented in (17):

(17) $$L_{t}^{\;}=L_{t}^{R}+L_{t}^{C}+L_{t}^{I},\;\forall t$$

Additionally, the reduced, off-peak tariff during certain hours of the day which is applied in Greece is taken into consideration. This means that this method not only maximizes the self-consumption of the smart grid but also, if it is necessary to import energy from the main grid, selects the hours which correspond to the reduced cost to do so. In this way, the overall cost of energy from the fuel-based main grid is reduced.

Furthermore, the proposed method is set to not exhaust one of its resources against another but is set to utilize the whole range of the resources available. This means that, if the RES production is not enough to cover the demand, the algorithm shall not choose to drain one source (e.g., the BESS) while leaving another source (e.g., the EVs) intact.

A critical point of this study is that, since the objective is to minimize the environmental impact of the smart grid’s operation, the optimizer is formulated in a way that all EVs and the BESS can only be charged from the smart grid’s RES.

Of note, if there was no capability for V2G operation the above model would be the same but with $D_{t}^{{\mathrm{EV}}_{i}}$ equal to zero throughout the day for all EVs.

A limitation of this methodology is that it takes into account the aggregations of DER. This is assumed because the smart grids to which this methodology may be applied are not as extensive as the large distribution grids. In this sense, effects such as branch losses, voltage angles, etc. can be ignored.

The proposed optimizer is implemented in Pyomo [47,48] and the solver used to solve the problem is the basic open-source nonlinear mixed integer programming (Bonmin) solver [49,50]. According to the basic algorithm of the solver, the problem constraints form a field of possible solutions (within the range of the decision variables). The set of decision variables that minimizes the value of the objective function is considered to be the optimal one. Therefore, it constitutes the optimal energy dispatch schedule.

### 2.2. Uncertainties Related to Day-Ahead Optimization

When it comes to day-ahead optimization, certain forecasts and assumptions need to be made regarding uncertainties, because not all of the parameters are initially known to the operator. In this sense, they may be useful for the planning of the system’s operation. Yet, no matter how accurate a forecast or an assumption can be, they may deviate from reality, which may affect the planning and operation of the system.

**Figure 2:**
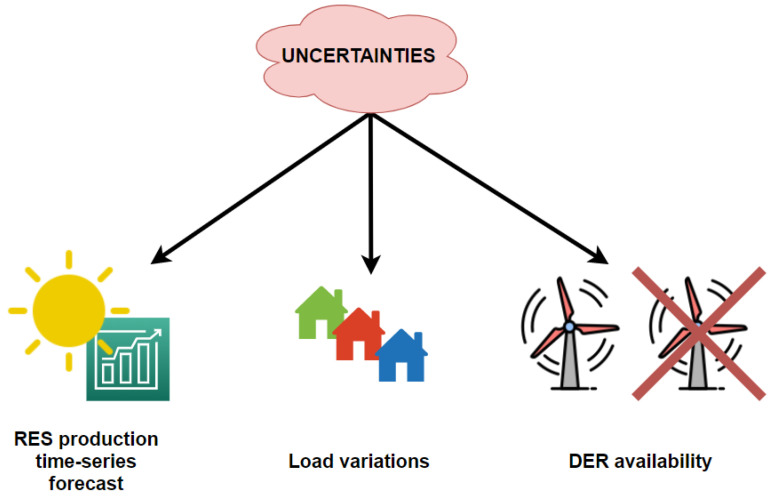
Uncertainties in day-ahead optimization.

**Figure 3:**
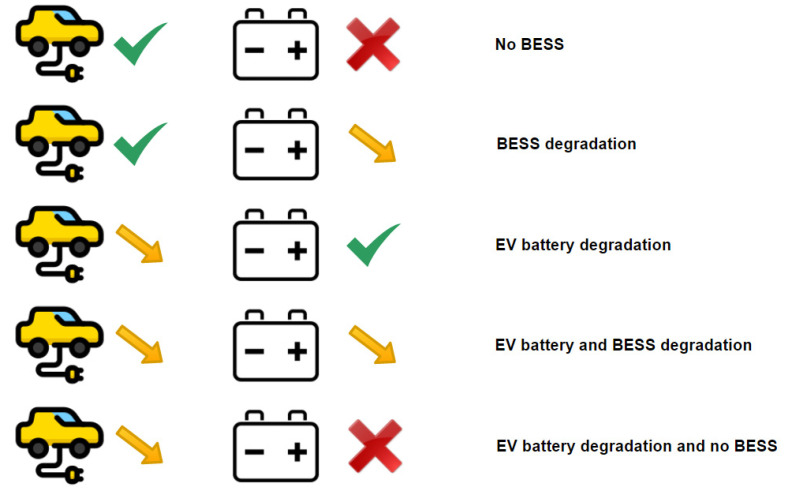
Loss and degradation scenarios of the smart grid’s storage systems (EVs in V2G mode and BESS).

In the case of smart grids, as presented in Figure 2, some of the most common uncertainties may be related to [33]:

- **RES production time-series forecast:** In the beginning of the day, a forecast of the PV and WF generation is produced so that the operator may proceed with the day-ahead optimization. Yet, these forecasted values come with respective errors. In order to evaluate the effect of the forecast errors in the system’s operation, the operator may consider the worst-case scenario that could occur. In this study, that would be to have the minimum possible PV and WF generation considering the maximum error throughout the whole forecast values. It should be noted that PV production and wind generation forecasts do not have the same errors because PV production is more predictable than wind generation [51,52]. In order to evaluate the worst-case scenario, the optimization problem should be solved considering (1), (3)–(18) instead of (2):
  (18) $$\left(P_{t}^{\mathrm{PV}}-e_{t}^{\mathrm{PV}}\right)\Delta_{t}+\left(P_{t}^{\mathrm{WF}}-e_{t}^{\mathrm{WF}}\right)\Delta_{t}+\underset{i}{\sum }(D_{t}^{{\mathrm{EV}}_{i}}\Delta_{t})+D_{t}^{\mathrm{BESS}}\Delta_{t}+I_{t}\Delta_{t}-E_{t}\Delta_{t}=L_{t}\Delta_{t}+\underset{i}{\sum }(C_{t}^{{\mathrm{EV}}_{i}}\Delta_{t})+C_{t}^{\mathrm{BESS}}\Delta_{t},\;\forall t$$
  where $e_{t}^{\mathrm{PV}}$ is the maximum PV forecast error and $e_{t}^{\mathrm{WF}}$ is the maximum wind generation error.
- **Load variations:** The total demand of a region usually comprises residential, commercial and industrial load. Out of all, the most unpredictable one is the residential load which depends on human activity and is not quite scheduled, in contrast with the operation of the commercial and industrial sector. The residential load is also highly related to thermal comfort, which increases its mutability and affects the overall load significantly [53]. In fact, it is estimated that 64% of the energy consumption in the residential sector is used for space heating, 15% is used for water heating, 14% is used for lighting and appliances and 6% is used for cooking [54]. In this study, the impact of the variations of the residential load are taken into consideration by simulating the expected results with special attention to the highest and lowest expected limits of residential load variations. In order to evaluate the different scenarios, the optimization problem should be solved considering (1)–(16) and (19) instead of (17):
  (19) $$L_{t}^{\;}{=L}_{t}^{R}{\;\pm \;v}_{t}{+L}_{t}^{C}{+L}_{t}^{I},\;\forall t$$
  where $v_{t}$ is the variation of the residential load at time-step t.
- **DER availability:** The smart grid operator may plan the day-ahead schedule considering that all of their assets shall be 100% available. However, sometimes this is not the case. More specifically, there are cases where a WT might stop functioning or the BESS may be unavailable due to fault or maintenance issues. Furthermore, the BESS or the batteries of the EVs may age through time and usage, which decreases their capacity. The impact of these issues to the overall performance of the system can be evaluated by the operator. In this study, the impact of DER availability is studied for (a) the PV system, for the whole range of 0–100% availability, (b) the WF, considering losing a number of WTs (i.e., losing 0, 1, …, all of the WTs), (c) the loss of the smart grid’s BESS, (d) the degradation (end of life-time) of the BESS, (e) the degradation of the batteries of the EVs, (f) the degradation of both the BESS and the batteries of the EVs, and (g) the loss of the BESS and degradation of batteries of the EVs.
  More specifically, the two-axis tracking PV system availability can be studied by gradually decreasing the overall PV production (from 0% decrease, i.e., normal operation, up to 100% decrease, i.e., complete failure of the installation). In order to evaluate the impact of the PV system’s availability, the optimization problem should be solved considering (1), (3)–(17) and (20) instead of (2), where $a^{\mathrm{PV}}$ is the PV availability ranging from (0%, 10%, …, 100%):
  (20) $$P_{t}^{\mathrm{PV}}a^{\mathrm{PV}}\Delta_{t}{+P}_{t}^{\mathrm{WF}}\Delta_{t}+\underset{i}{\sum }{(D}_{t}^{{\mathrm{EV}}_{i}}\Delta_{t}{)+D}_{t}^{\mathrm{BESS}}\Delta_{t}{+I}_{t}\Delta_{t}{\;-\;E}_{t}\Delta_{t}{=L}_{t}\Delta_{t}+\underset{i}{\sum }{(C}_{t}^{{\mathrm{EV}}_{i}}\Delta_{t}{)+C}_{t}^{\mathrm{BESS}}\Delta_{t},\;\forall t$$
  The WF availability can be studied by decreasing the total number of operational WTs. This can be accomplished by considering having initially all WTs available, then having all WTs available except from one (and so on) until having no WTs available. In order to evaluate the impact of the WF’s availability, the optimization problem should be solved considering (1)–(15), (17) and (21) instead of (16), where n is the number of unavailable WTs and its values may be in the range of (0, 1, …, total number of WTs):
  (21) $$P_{t}^{\mathrm{WF}}=\overset{\mathrm{WTs}-n}{\underset{\;}{\sum }}P_{t}^{{\mathrm{WT}}_{w}},\;\forall t$$
  As regards the loss or degradation of the smart grid’s storage, including the BESS and the EVs, a variety of combinations can be studied, as presented in Figure 3.

In order to evaluate the impact of the loss of the smart grid’s BESS, the optimization problem should be solved considering (1), (3)–(10), (16), (17) and (22) instead of (2):

(22) $$P_{t}^{\mathrm{PV}}\Delta_{t}{+P}_{t}^{\mathrm{WF}}\Delta_{t}+\underset{i}{\sum }{(D}_{t}^{{\mathrm{EV}}_{i}}\Delta_{t}{)+I}_{t}\Delta_{t}{\;-\;E}_{t}\Delta_{t}{=L}_{t}\Delta_{t}+\underset{i}{\sum }{(C}_{t}^{{\mathrm{EV}}_{i}}\Delta_{t}),\;\forall t$$

In order to evaluate the degradation of the BESS, the optimization problem should be solved considering (1)–(17) with special attention to (11) where the capacity is reduced to 80% of the nominal, according to [55,56]. Similarly, for the evaluation of the impact of the degradation of the batteries of the EVs, the optimization problem should be solved considering (1)–(17) with special attention to (3) where the capacity is reduced to 80% of the nominal. Of course, the two degradation scenarios can be combined, and their impact can be evaluated by solving the optimization problem considering (1)–(17) and 80% capacity in both (3) and (11). Finally, in order to have all the combinations examined, it is important to evaluate the impact of the scenario where the BESS does not operate and the batteries of the EVs are degraded. This can be studied by solving the optimization problem considering (1), (3)–(10), (16), (17) and (22), with 80% capacity in (3).

## 3. Case Study

The optimization method described above is tested on a hypothetical smart grid located in Greece. The overall daily demand of the smart grid contains a mix of residential, commercial and industrial load curves, attained from the CIGRE benchmark systems, presented in [57]. 

The smart grid’s assets include a two-axis tracking PV system, WTs and a BESS, as presented in Table 1, but also EVs (with V2G capability) as presented in Table 2. In more detail, the smart grid is equipped with a two-axis tracking PV system with nominal power equal to 1 MW, which is justified by the smart grid’s needs. In fact, the total average daily load of the CIGRE benchmark system is equal to 8.8 MWh and the average daily production of the two-axis tracking PV system is equal to 5.7 MWh. The smart grid is also equipped with 2 Bonus B23/150 WTs [58]. Each WT’s installed power is equal to 150 kW, its hub height is equal to 24.5 m and its average daily production is equal to 0.9 MWh. The selection of the installed number of WTs is based on the EU goal of having 24% of the demand covered by WTs [59]. It is assumed that the smart grid is equipped with adequate RES in order to showcase the benefits of the proposed methodology. If there was lack of renewable production (either due to the location/potential of the smart grid or due to lack of adequate RES installations) there would be no reason to study the environmental-friendly energy management (as the BESS and V2G mode would not be utilized and the RES production would directly feed the load).

The BESS of the smart grid has capacity equal to 1 MWh, in order to be compatible with the smart grid’s RES. Additionally, the maximum power that can be charged to/discharged from it is equal to 500 kW and its efficiency is equal to 0.92, as presented in [60]. Furthermore, it is assumed that the back-up energy required in case of an emergency corresponds to a minimum state of charge equal to 50%, since the average hourly demand of this use case is equal to 367 kWh [46]. 

As regards the EVs, they are modeled according to Nissan Leaf’s technical specifications [61], presented in Table 2. More specifically, the nominal battery energy is equal to 40 kWh and the maximum input and output power is equal to 3.6 kW. The consumption due to transportation is 164 Wh/km and the battery’s efficiency when the EV is being charged or discharged is equal to 0.9.

The features and behavior of the EV owners are presented in Table 3. It is assumed that each EV owner travels 5.6 km daily according to [62], which is the average daily travelling distance in Greece. It is calculated that the energy consumption due to the daily transportation is equal to 918.4 Wh according to (23), where ${\mathrm{DC}}^{{\mathrm{EV}}_{i}}$ is the daily consumption of an EV, $d^{{\mathrm{EV}}_{i}}$ is the distance it travels and $c^{{\mathrm{EV}}_{i}}$ is its consumption per kilometer.

(23) $${\mathrm{DC}}^{{\mathrm{EV}}_{i}}{=d}^{{\mathrm{EV}}_{i}}c^{{\mathrm{EV}}_{i}},\;\forall i$$

The overall daily travelling distance of each EV is assumed to be divided in two equal parts, in order for the EV owners to arrive and to leave their workplace respectively. In this sense, $T_{t}^{{\mathrm{EV}}_{i}}$, is equal to 459.2 Wh. The time of arrival at the EV owners’ workplace follows uniform distribution from 7:00 am until 9:00 am, which is a representative interval according to [63]. This means that for a total of 300 EV owners, 100 arrive at their workplace at 7:00, 8:00 and 9:00 equally. It is also assumed that the EV owners remain at their workplace for 8 h and expect to have the batteries of their EVs fully charged by the time they leave it. 

Finally, it is taken into account that the reduced electricity price in Greece from the 1st of May until the 31st of October (summer period) lasts from 23:00 until 7:00, while from the 1st of November until the 30th of April (winter period) the reduced cost per kWh lasts from 2:00 until 8:00 and from 15:00 until 17:00 [45].

**Figure 4:**
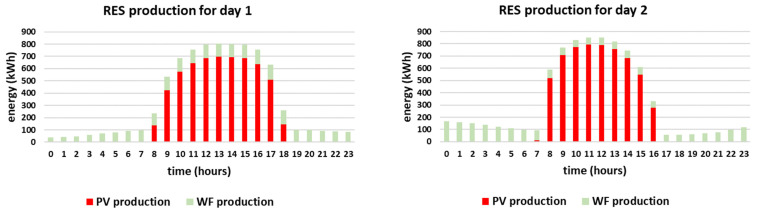
PV and WF generation curves.

Due to the fact that there are two different policies for reduced cost per kWh, depended on the month of the year, two representative days of the year need to be simulated, one for each policy, i.e., simulated day 1 and simulated day 2. As presented in Table 4, simulated day 1 is considered to be a representative day because the PV production is equal to 5.8 MWh, which is close to the daily average (5.7 MWh) and the WF production is equal to 2.2 MWh, which is 25% of the daily demand and meets the EU goal of 24% [59]. Furthermore, since this day occurs in October, the electricity price is reduced from 23:00 until 7:00. On the other hand, simulated day 2 is considered to be a representative day because the PV production is equal to 5.9 MWh, which is also close to the daily average of 5.7 MWh and the WF production is equal to 2.1 MWh, which is 24% of the daily demand and equal to the EU goal. Since this representative day occurs in January, the cost per kWh is reduced from 2:00 until 8:00 and then again from 15:00 until 17:00. It should be noted that the two selected days differ from each other in terms of weather conditions as October is considered to be a warm month in Greece (typical of a mild beginning of autumn in such a Mediterranean Southeastern European country), whereas January is considered to be a cold month (heart of wintertime). The PV and wind generation curves are attained from [64,65] and are presented in Figure 4. 

Apart from the main/validity analysis of the case study (which will be conducted according to the aforementioned boundaries), in order to evaluate the effect of certain parameters to the proposed solution an extensive sensitivity analysis is carried out, as presented in Section 2.2. In this context, the effect of the forecast error of RES production time-series needs to be evaluated (in this case the error concerns the PV forecast and WF forecast). Additionally, the effect of residential load variations needs to be examined. Last but not least, the sensitivity analysis presents the effect of DER availability, including PV panels, WTs, BESS, and EVs.

## 4. Results

This Section aims to present the results of the case study as well as the results of the sensitivity analysis.

### 4.1. Case Study Results

**Figure 5:**
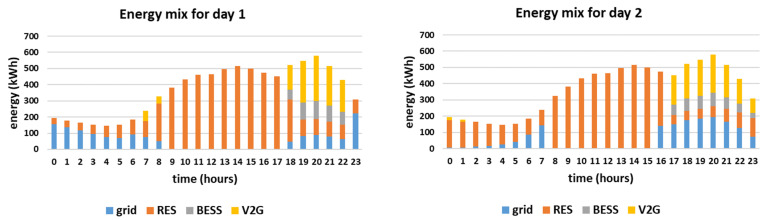
Hourly energy mixes for the two simulated days.

**Figure 6:**
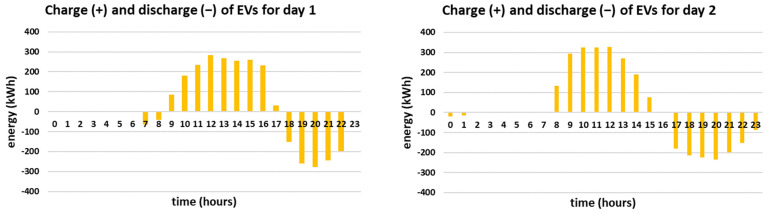
Charge and discharge (V2G) of EVs for the two simulated days.

**Figure 7:**
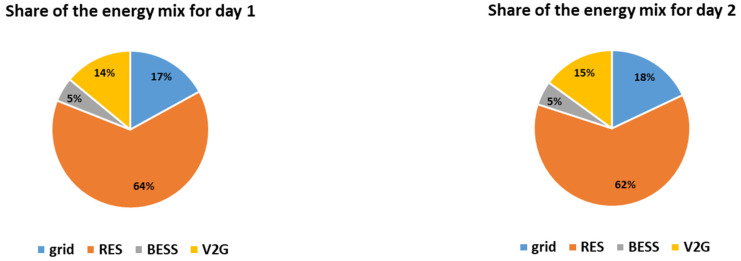
Share of the energy mix for the two simulated days.

The hourly energy mixes for the two simulated days are presented in Figure 5. Furthermore, the charge/discharge schedules of the EVs are presented in Figure 6 and the overall daily energy mix of each simulated day is presented in Figure 7.

It is noted that in both simulated days the energy mix relies mostly on RES production feeding directly the load, i.e., 64% and 62% respectively. The RES feed directly the load throughout the day, especially during noon when the PV production is high, as presented in Figure 5. During the hours when the RES production exceeds the demand, i.e., during 9:00–17:00 for day 1 and during 8:00–15:00 for day 2, the EVs are charged since the objective is to maximize the smart grid’s self-consumption, according to Figure 6. In both cases, the EVs contribute to the energy mix (V2G) when the RES production is lower than the demand, especially in the afternoon, having a substantial contribution to the energy mix, i.e., 14% and 15% respectively. The BESS is in both cases fully utilized, contributing 5%, based on the minimum state of charge set equal to 50% for back-up (which means that 0.5 MWh of the energy stored inside the BESS is kept as back-up). Additionally, it is noted that the reduced grid price timing is taken into account in both simulated days, as the smart grid uses only energy imported from the main grid if the RES production does not meet the demand in the time intervals when the reduced grid cost per kWh applies. Altogether, even though the two cases have different production curves, the overall share of the energy mix is almost the same as the storage units (EVs and BESS) distribute the RES production efficiently throughout the day, proving the effectiveness of the proposed methodology. However, it should be mentioned that the percentage of RES production feeding directly the load is slightly higher (64% compared to 62%) and the percentage of V2G is slightly lower (14% compared to 15%) for day 1, even though both days are chosen to have equal RES production (i.e., 8 MWh). This is attributed to the fact that the RES production for day 1 is more evenly distributed throughout the day than in day 2. For example, according to Figure 4, at 8:00 the RES production for day 2 is more than twice as much as the RES production for day 1. As a result, in day 1 more energy feeds directly the load and less energy needs to be stored (as it exceeds the demand). Furthermore, since the storage units (EVs and BESS) are only charged by RES and are not ideal, meaning that they have efficiency rate lower than 1, the energy losses need to be covered from the main grid. This is the reason why in day 2 the contribution of the main grid is equal to 18% while in day 1 is equal to 17%, as stated in Figure 7.

### 4.2. Sensitivity Analysis Results

This sub-section aims to present the results of the extensive sensitivity analysis that was carried out according to the methodology of Section 2.2.

#### 4.2.1. Sensitivity Analysis Results Considering the RES Production Time-Series Forecast

**Figure 8:**
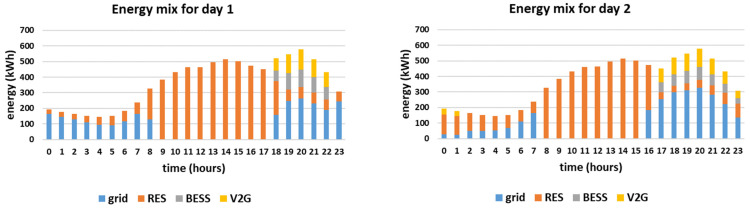
Hourly energy mixes considering the worst-case scenario for the RES production time-series forecast for the two simulated days.

**Figure 9:**
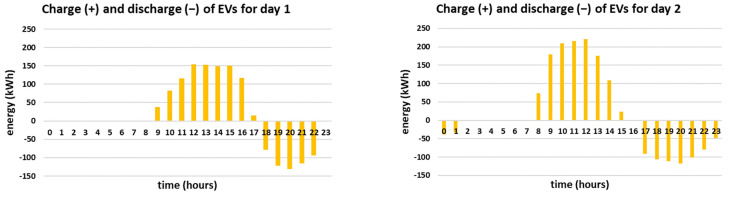
Charge and discharge (V2G) of EVs for the two simulated days considering the worst-case scenario for the RES production time-series forecast.

**Figure 10:**
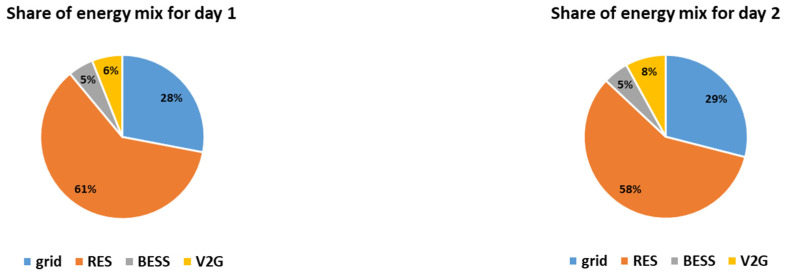
Share of the energy mix for the two simulated days considering the worst-case scenario for the RES production time-series forecast.

The hourly energy mixes for the two simulated days, considering the worst-case scenario for the RES production are presented in Figure 8. The corresponding charge/discharge schedules of the EVs and the overall daily energy mix of each simulated day are presented in Figure 9 and Figure 10, respectively. It should be noted that the PV forecast error for time-step t, $e_{t}^{\mathrm{PV}}$, is considered to be equal to 11%*$P_{t}^{\mathrm{PV}}$, according to [51]. On the other hand, the wind generation forecast error for time-step t, $e_{t}^{\mathrm{WF}}$, is considered to be equal to 24.67%*$P_{t}^{\mathrm{WF}}$, according to [52]. Table 5 compares the day-ahead optimization results with/without V2G, with/without uncertainty regarding the RES production time-series forecast.

As expected, the RES production feeding directly the load is lower than in the reference cases, i.e., 61% and 58% of the total energy mix, respectively (compared to 64% and 62% respectively for the reference cases). Furthermore, since the EVs are only charged from the RES, V2G is significantly reduced, i.e., 6% and 8% of the total energy mix (compared to 14% and 15% respectively for the reference cases), as presented in Figure 9 and Figure 10. Since the storage can only be charged by the RES and the RES production is reduced, the smart grid’s demand is met through fuel-based energy imported from the main grid, as presented in Figure 8, which is increased reaching up to 29% of the daily consumption.

Yet, either with or without taking the RES uncertainty into consideration, it should be noted that the smart grid with V2G capability has self-consumption rate at least equal to 71%, reaching up to 83% and there is no reverse flow of energy, according to Table 5. The maximum V2G contribution is considered to be equal to 1324 kWh, which corresponds to 15% of the smart grid’s load. On the contrary, without V2G capability, self-consumption cannot exceed 69% and reverse flow of energy is observed in all cases, reaching up to 1504 kWh, highlighting the importance of EVs as storage units.

#### 4.2.2. Sensitivity Analysis Results Considering Residential Load Variations

**Figure 11:**
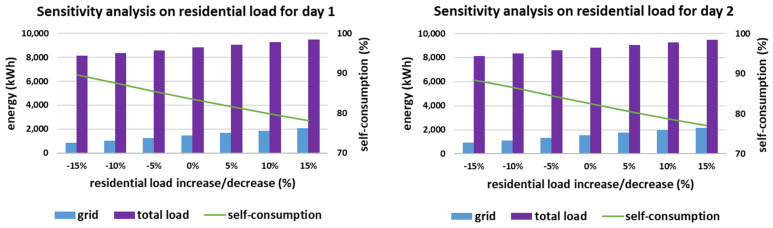
Sensitivity analysis on the residential load for the two simulated days.

The impact of the variations of the residential load is presented in Figure 11. The variation at time-step t, $v_{t}$, is considered to range from −15%*$L_{t}^{R}$ up to +15%*$L_{t}^{R}$, with a step set equal to 5%, according to [66]. The robustness of the proposed methodology is proven by the self-consumption rate which is in all cases higher than 77%. This value corresponds to 15% increase of residential load for day 2. Of note, self-consumption may even reach 89.7%, which occurs for −15% residential load for day 1. 

#### 4.2.3. Sensitivity Analysis Results Considering DER Availability

**Figure 12:**
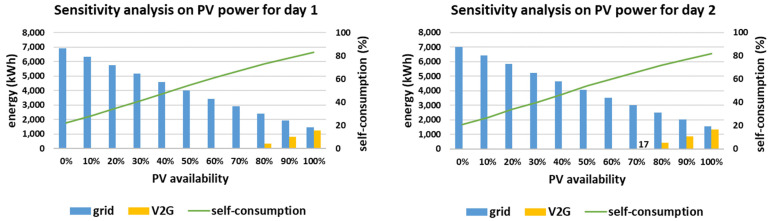
Sensitivity analysis on PV availability for the two simulated days.

The sensitivity analysis regarding the two-axis tracking PV system availability is presented in Figure 12, considering the whole range of 0–100% with step equal to 10%. In this case, the smart-grid’s self-consumption may be affected substantially, with minimum value equal to 21%, for day 2 considering 0% PV availability. This happens because the smart grid under study is mostly based on PV production, as presented in Figure 4. The V2G capability is utilized for PV availability at least equal to 80% in case of day 1 and for PV availability at least equal to 70% for day 2 (with a small contribution equal to 17 kWh). This small inclination between the starting points of V2G usage has been expected, as V2G energy has always been higher for day 2 so far (compared to day 1) and is attributed to the RES production profile of day 2, as previously discussed. 

**Figure 13:**
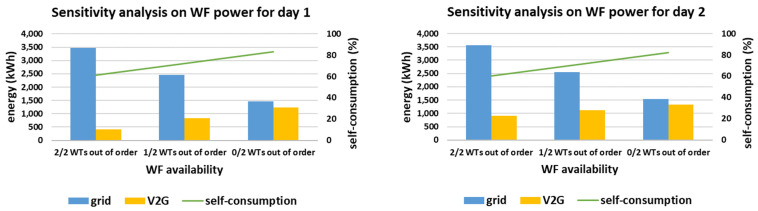
Sensitivity analysis on WF availability for the two simulated days.

The sensitivity analysis regarding the WF availability is presented in Figure 13, considering the possible loss of the smart grid’s WTs. Since the WF production is not as high as the PV production for both simulated days (see Table 4), the WF availability has a lower effect on the smart grid’s self-consumption. In fact, even without wind generation, i.e., considering 2/2 WTs out of order, the self-consumption is at least equal to 60% (observed for day 2). Additionally, in all cases of WF availability, the overall RES production is enough to enable V2G contribution, ranging from 419 kWh (2/2 WTs out of order for day 1) up to 1324 kWh (0/2 WTs out of order for day 2).

**Figure 14:**
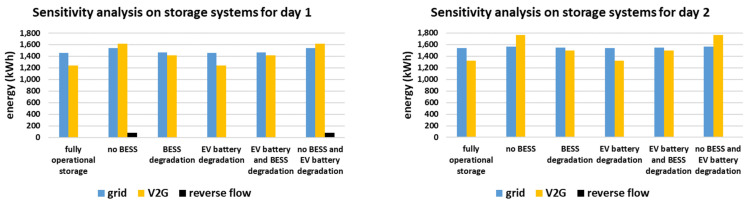
Sensitivity analysis on the smart grid’s storage systems (EVs in V2G mode and BESS) for the two simulated days.

The sensitivity analysis on the smart grid’s storage systems is presented in Figure 14. Five main combinations of absence or degradation of both storage systems, i.e., EVs and BESS, are considered. It should be noted that in all cases the self-consumption of the smart grid remains almost the same, i.e., equal to 83% for day 1 and 82% for day 2. This happens because the overall system capacity is enough to perform the energy management efficiently. 

More specifically, when considering only EV battery degradation, the results remain the same as in the reference cases, where fully operational storage is assumed (same self-consumption, same V2G energy usage, same amount of energy from the main grid). This occurs as the energy utilized from the batteries of the EVs is low enough to not be affected by degradation, i.e., 1237 kWh for day 1 and 1324 kWh for day 2 while the overall battery energy of EVs is equal to 12,000 kWh. 

However, since the BESS is fully utilized in the reference cases, in the scenarios where BESS degradation is considered (with or without simultaneous degradation of the batteries of the EVs) the energy mix is affected. In fact, since the BESS can no longer fully participate due to degradation, the energy it was supposed to store gets stored in the EVs. As a result, more V2G energy is used, i.e., equal to 1413 kWh for day 1 and 1500 kWh for day 2. 

The energy mix is further affected when considering the absence of the BESS, either with or without EV battery degradation. In this case, the EVs need to absorb even more energy and contribute more to the energy mix, as presented in Figure 14, i.e., 1615 kWh for day 1 and 1764 kWh for day 2. Yet, as presented in the respective result for day 1, there is a small amount of energy, equal to 77 kWh, flowing reversely. This reverse flow is observed during afternoon hours and is attributed to the profile of RES production in combination with the constraints of the EVs imposed by their users. In fact, the EVs depart from the workplace at 15:00, 16:00 and 17:00. At this time, they are expected to be fully charged and travelling towards the residences of the EV owners. However, at that time interval, i.e., 15:00–17:00, the RES production happens to be particularly high, reaching up to 800 kWh hourly, as presented in Figure 4. Since the RES production is high and the EVs are expected to be fully charged, there is not enough storage capability for all the RES production. Therefore, a small amount of RES production flows reversely to the main grid. This would have been avoided if the BESS was available and showcases its importance.

## 5. Conclusions

This paper presents a day-ahead optimization method for the optimal integration of V2G capability in smart grids considering a rich energy mix, taking advantage of the possibilities and information provided by the smart grid’s sensors and communication system. The aim of the proposed optimizer is the minimization of the smart grid operation’s environmental footprint. For this purpose, the EVs as well as the BESS of the smart grid are charged with energy generated from RES, while the energy imported from the (considered as fuel-based) main grid is minimized. Additionally, attention is paid to the intervals where reduced grid cost per kWh is applied and to the BESS, which cannot be fully discharged as it may be used for back-up in case of an emergency. The simulations are carried out in a hypothetical smart grid located in Greece for two representative days of the year with different reduced grid price intervals. The results highlight the merits of V2G capability, reaching:

- Self-consumption at least equal to 82%
- V2G energy usage up to 15%.

Furthermore, various parameters of the method are taken into account in this study, including:

- The forecast error of the RES production time-series, which may reduce the self-consumption down to 71%
- The residential load variations which may reduce the self-consumption down to 77%
- The RES and storage availability, which may reduce the self-consumption even down to 21% (in the case of total loss of the PV system).

Overall, the sensitivity analysis results highlight the effect of selected parameters in the smart grid’s self-consumption and the importance of V2G participation in the energy mix.

## Figures and Tables

**Table 1:** Specifications of the PV, WT and BESS utilized in the case study.

Asset	Magnitude	Value
Two-axis tracking PV system	Installed power	1 MW
	Average daily production	5.7 MWh
WT	Installed power of one WT	150 kW
	Hub height	24.5 m
	Average daily production per WT	0.9 MWh
	Number of WTs	2
BESS	Nominal energy	1 MWh
	Maximum input/output power	500 kW
	Efficiency	0.92

**Table 2:** EV specifications.

EV Technical Specification	Value
Nominal battery energy	40 kWh
Maximum input/output power	3.6 kW
Consumption	164 Wh/km
Efficiency	0.9

**Table 3:** EV owner characteristics.

EV Owner Feature/Behavior	Value
Daily travelling distance	5.6 km
Daily consumption due to transportation	918.4 Wh
User defined state of charge at departure from workplace	100%
Time of arrival at workplace	7:00–9:00 following uniform distribution
Working hours	8 h
Number of EV owners	300

**Table 4:** Data of the simulated days.

Data	Simulated Day 1	Simulated Day 2
Time interval for reduced cost per kWh	23:00–7:00	2:00–8:00 and 15:00–17:00
PV production	5.8 MWh	5.9 MWh
WF production	2.2 MWh	2.1 MWh

**Table 5:** Comparison between the day-ahead optimization modes with/without V2G, with/without uncertainty regarding the RES production time-series forecast.

Operation Mode	Self Consumption (%)		Energy from the Grid (kWh)		Energy from the EVs (kWh)		Reverse Flow of Energy (kWh)	
	Day 1	Day 2	Day 1	Day 2	Day 1	Day 2	Day 1	Day 2
V2G	83%	82%	1456	1541	1237	1324	0	0
V2G with RES uncertainty	72%	71%	2479	2568	541	728	0	0
Without V2G	69%	67%	2693	2865	0	0	1382	1504
Without V2G with RES uncertainty	66%	63%	3020	3297	0	0	596	827

## Data Availability

Data sharing is not applicable to this article.

## Abbreviations

**Indices**	
i	EV indice
t	Time-step indice
w	WT indice
**Parameters**	
$a^{\mathrm{PV}}$	PV availability
$c^{{\mathrm{EV}}_{i}}$	Consumption per kilometer of the i-th EV
$C_{\max}^{{\mathrm{EV}}_{i}}$	Maximum power that can be charged to the i-th EV
$C_{\max}^{\mathrm{BESS}}$	Maximum power that can be charged to the BESS
$d^{{\mathrm{EV}}_{i}}$	Daily distance travelled by the i-th EV
${\mathrm{DC}}^{{\mathrm{EV}}_{i}}$	Daily consumption of the i-th EV
$D_{\max}^{{\mathrm{EV}}_{i}}$	Maximum power that can be discharged from the i-th EV
$D_{\max}^{\mathrm{BESS}}$	Maximum power that can be discharged from the BESS
$e_{t}^{\mathrm{PV}}$	Maximum PV forecast error at time-step t
$e_{t}^{\mathrm{WF}}$	Maximum WF forecast error at time-step t
$L_{t}^{\;}$	Load at time-step t
$L_{t}^{C}$	Commercial load at time-step t
$L_{t}^{I}$	Industrial load at time-step t
$L_{t}^{R}$	Residential load at time-step t
$P_{t}^{{\mathrm{WT}}_{w}}$	Production of the w-th WT at time-step t
$P_{t}^{\mathrm{PV}}$	Production of the PV system at time-step t
$P_{t}^{\mathrm{WF}}$	Production of the WF at time-step t
${\mathrm{SOC}}_{\max}^{{\mathrm{EV}}_{i}}$	Maximum state of charge of the i-th EV
${\mathrm{SOC}}_{\max}^{\mathrm{BESS}}$	Maximum state of charge of the BESS
${\mathrm{SOC}}_{\min}^{{\mathrm{EV}}_{i}}$	Minimum state of charge of the i-th EV
${\mathrm{SOC}}_{\min}^{\mathrm{BESS}}$	Minimum state of charge of the BESS
${\mathrm{Tdep}}_{i}$	Time of departure of the i-th EV
$T_{t}^{{\mathrm{EV}}_{i}}$	Consumption of the i-th EV due to transportation at time-step t
${\mathrm{USOC}}_{\;}^{{\mathrm{EV}}_{i}}$	User-defined state of charge of the i-th EV at the time of departure
$v_{t}$	Variation of residential load at time-step t
$\eta^{{\mathrm{EV}}_{i}}$	Efficiency of the battery of the i-th EV
$\eta^{\mathrm{BESS}}$	Efficiency of the BESS
Δt	Duration of time-step t
**Decision variables**	
$C_{t}^{{\mathrm{EV}}_{i}}$	The power charged to the i-th EV at time-step t
$C_{t}^{\mathrm{BESS}}$	The power charged to the BESS at time-step t
$D_{t}^{{\mathrm{EV}}_{i}}$	The power discharged from the i-th EV at time-step t
$D_{t}^{\mathrm{BESS}}$	The power discharged from the BESS at time-step t
$E_{t}$	The power exported to the main grid at time-step t
$I_{t}$	The power imported to the main grid at time-step t
${\mathrm{SOC}}_{t}^{{\mathrm{EV}}_{i}}$	State of charge of the i-th EV at time-step t
${\mathrm{SOC}}_{t}^{\mathrm{BESS}}$	State of charge of the BESS at time-step t
$u_{{\mathrm{ch}}_{t}}^{{\mathrm{EV}}_{i}}$	Binary variable indicating whether the i-th EV is being charged at time-step t
$u_{{\mathrm{ch}}_{t}}^{\mathrm{BESS}}$	Binary variable indicating whether the BESS is being charged at time-step t
$u_{{\mathrm{dch}}_{t}}^{\mathrm{BESS}}$	Binary variable indicating whether the BESS is being discharged at time-step t
$u_{{\mathrm{dch}}_{t}}^{{\mathrm{EV}}_{i}}$	Binary variable indicating whether the i-th EV is being discharged at time-step t
